# Using DNA microarrays to study host-microbe interactions.

**DOI:** 10.3201/eid0605.000511

**Published:** 2000

**Authors:** C A Cummings, D A Relman

**Affiliations:** Stanford University, Stanford, California, USA. cummings@cmgm.stanford.edu

## Abstract

Complete genomic sequences of microbial pathogens and hosts offer sophisticated new strategies for studying host-pathogen interactions. DNA microarrays exploit primary sequence data to measure transcript levels and detect sequence polymorphisms, for every gene, simultaneously. The design and construction of a DNA microarray for any given microbial genome are straightforward. By monitoring microbial gene expression, one can predict the functions of uncharacterized genes, probe the physiologic adaptations made under various environmental conditions, identify virulence-associated genes, and test the effects of drugs. Similarly, by using host gene microarrays, one can explore host response at the level of gene expression and provide a molecular description of the events that follow infection. Host profiling might also identify gene expression signatures unique for each pathogen, thus providing a novel tool for diagnosis, prognosis, and clinical management of infectious disease.


### Microarray Data Analysis

Microarrays are likely to become a standard tool of the microbiology laboratory. However, because genomewide datasets are large and comprehensive, analysis of an experiment can become daunting. Careful experimental design can simplify analysis and interpretation of the dataset by minimizing the number of variables that affect gene expression. For example, strain differences can be minimized by using isogenic mutants, tissue complexity can be reduced by studying clonal cell lines, and complex regulatory pathways can be tamed by experimental modulation of transgene expression (6).

Because microarray experiments result in such large amounts of data, false-positive results are likely. Analyzing multiple independent experiments may eliminate spurious results (32). Also important is validation of differentially expressed genes by independent methods. When checked by a number of methods including quantitative RT-PCR (6, 35), Northern blotting (33, 34, 36), and protein expression (33, 34), most differentially expressed genes have been confirmed. For example, 72 of 72 mRNAs found to be regulated in response to cytomegalovirus (CMV) infection were confirmed by either prior reports or Northern blotting (37). Future challenges for microarray researchers will include developing databases and algorithms to manage and analyze vast genomic-scale datasets.

### Image Analysis Software

The first step after hybridization is capturing an image of the array and from it, extracting numerical data for each element (Figure 1). Several software applications, including those packaged with most commercial scanners, can perform this task. However, not all programs use the same algorithms to calculate signal intensity, and each of the programs exports a different constellation of signal quality measurements, complicating comparisons between data acquired with different applications (38). If gene expression datasets are to be compared, these measurements must be standardized. Furthermore, standard, robust statistical methods must be developed for assigning significance values to gene expression measurements.

### Databases

Although many laboratories are now capable of collecting microarray data, few have access to a database that can effectively meet their data requirements. With considerable investment of resources, a few full-featured, relational gene expression databases have been developed, but these are not available for public deposition of data (e.g., http://genome-www4.stanford.edu/MicroArray/MDEV/index.html; http://www.nhgri.nih.gov/DIR/LCG/15K/HTML/dbase.html). Recently released, the freely available AMAD software package (http://www.microarrays.org/software.html) provides basic microarray data storage and retrieval capabilities to the average laboratory.

A grander goal for the community is establishing a consolidated resource for public distribution of microarray data (39-41). Again, the lack of a standard format for microarray data interferes with creating such a resource (38,39). The European Bioinformatics Institute, recognizing this obstacle, has proposed defining a standard based upon XML, a computer markup language that combines data and formatting in a single file for distribution over the World-Wide Web (40; http://www.ebi.ac.uk/arrayexpress/).

### Algorithms

Inferring biologically meaningful information from microarray data requires sophisticated data exploration. Most global gene expression analyses have used some form of unsupervised clustering algorithm (16,42-44) to find genes coregulated across the dataset (Figure 1). A primary justification for this approach is that shared expression often implies shared function (38,43). In datasets containing many experiments, clustering can also group experiments on the basis of gene expression profiles, an approach that has been successful in classifying tumor-derived cell lines (19, 45) and tumor subtypes (12-17).

When a coregulated class of genes is known, supervised clustering algorithms, which are trained to recognize known members of the class, can assign uncharacterized genes to that class. For example, a machine-learning method known as a support vector machine has been used to classify yeast genes by function on the basis of shared regulation (46). Robust determination of coregulated gene clusters may be achieved by using a tiered approach: unsupervised clustering to identify coregulated genes followed by testing and refinement with supervised algorithms (47).

Although clustering algorithms will continue to be a mainstay in the analysis of gene expression datasets, a wealth of other data-mining techniques have yet to be applied (38,48). Preliminary reports indicate that many algorithms and visualization methods are being developed, but their ability to extract biologic insight has yet to be established (49-51).

The study of microbial pathogens, and prokaryotes in general, will require the development of some specialized analysis tools. First, the compact and modular structure of prokaryotic genomes—and in particular, the presence of operons and pathogenicity islands—suggests that important insights may be gained by mapping gene expression information onto genomic structure. In addition, because gene expression will be measured in many different pathogens, often under the same environmental conditions, tools for cross-species comparison of gene expression data will permit the detection of conserved transcription responses.

### Examining a Microorganism: Application of DNA Microarrays

Microarray technology promises to speed the study of uncharacterized or poorly characterized microbes by contributing to annotation of the microbial genome, enabling exploration of microbial physiology, and identifying candidate virulence factors.

### Designing a Microbial Genome Microarray

Designing a whole-genome DNA microarray for a fully sequenced microbe is conceptually straightforward. Several sensitive microbial gene-finding programs can quickly and accurately predict most ORFs (52-57). DNA fragments representing each of the ORFs can be obtained by PCR amplification that uses ORF-specific oligonucleotides, the design of which can be automated with primer design software such as Primer3 (58). Homology-searching algorithms should be used to choose regions of genes that will not cross-hybridize with other regions of the genome. After a simple purification step, PCR fragments can be arrayed by a robotic arrayer (5). This basic approach has been used to construct a 4,290-ORF *E. coli* microarray (10, 11) and a 3,834-ORF *Mycobacterium tuberculosis* microarray (30) as well as full-genome arrays for *Helicobacter pylori* (S. Falkow, pers. comm.) and *Caulobacter crescentus* (L. Shapiro, pers. comm.). Microarray fabrication based on photolithographic synthesis of oligonucleotides in situ is also a viable approach and has been successfully used for the production of an *E. coli* complete ORF chip (*E. coli* Genome Array, Affymetrix, Santa Clara, CA).

The utility of microarrays is not restricted to fully sequenced organisms. A powerful screening tool can be obtained by arraying DNA libraries, as has been done for the eukaryotic pathogen, *Plasmodium falciparum* (59). A DNA microarray of 3,648 random genomic clones was used to identify >50 genes for which expression differed significantly between the trophozoite and gametocyte stages. The major limitation of this approach is that the identity of any element of interest must be determined after the experiment.

### Annotating the Function of a Microbial Genome

For many pathogens, the number of genes for which function information is available is usually low. Moreover, the relative insufficiency of genetic tools can make obtaining such information difficult. However, because >70% of bacterial proteins have orthologs in other organisms (60,61), one can leverage extensive knowledge of function from the model organisms to infer function for a pathogen's genome. Similarity searches alone will predict functions of many genes.

We expect the study of genomewide expression patterns to contribute even further to annotation of function. The rationale for this belief follows from the observation that shared expression often implies shared function (38). As suggested by Brown and Botstein (21), the inclusion of a gene with a characterized ortholog in a coregulated gene cluster can predict the function of the remaining genes in that cluster, thus bootstrapping the function annotation of the pathogen's genome. This assertion is borne out in a study of global gene expression in *Saccharomyces cerevisiae*. Clustering of 2,467 gene expression profiles across a series of 78 experiments representing eight cellular processes demonstrated coregulation of genes that participated in shared cellular function (43). Therefore, the acquisition of a pathogen's gene expression data from even a modest number of experimental conditions may lead to testable hypotheses about function for a substantial number of genes, even those lacking sequence similarity to genes whose function has been characterized.

### Probing a Microbe's Physiologic State

The assumption that genes are preferentially expressed when their function is required allows inference of gene function directly from physiologic gene response. For example, genes preferentially transcribed during the diauxic shift in yeast are predicted to contribute in the metabolic transition to respiration (9). Thus, gene expression studies will contribute to function annotation by identifying the specific environmental and physiologic conditions in which each gene is expressed. Furthermore, as annotation improves, the direction of this inference may be reversed, i.e., if information on function is known for many genes, genomic expression profiling may reveal the physiologic state of the organism.

Two studies have used whole-genome DNA arrays to explore gene expression response to environmental stimuli in *E. coli*. First, treatment with isopropyl-ß-D-thiogalactopyranoside (IPTG) was shown to induce only the *lac* operon, and to a lesser extent, the melibiose operon (11). In a second study, comparison of strains grown in minimal versus rich media revealed 344 genes that were differentially expressed between the two conditions: preferential expression of the translation apparatus in rich media and the amino acid biosynthetic pathways in minimal media were entirely consistent with prior data (10). Finally, examination of gene expression during heat shock revealed 119 genes with altered expression levels, all but 35 of which were previously recognized as heat shock genes (11). These studies confirm that the physiologic state of bacteria can be inferred from gene expression data.

In the first report of global gene expression monitoring in a bacterial pathogen, oligonucleotide microarrays were used to measure the relative transcript levels of 100 *Streptococcus pneumoniae* genes during the development of natural competence and during stationary phase (29). The results confirmed induction of the *cin* operon and identified 11 genes differentially regulated in stationary versus exponential phase. Of course, gene expression monitoring is not restricted to the study of bacterial pathogens. Transcription of the CMV genome was measured during infection by using an array of 75-mer oligonucleotides representing each of the 226 predicted CMV ORFs (62). By blocking translation or DNA replication, the researchers revealed a detailed classification of CMV genes into four kinetic classes, in agreement with previous reports, and assigned many ORFs, for which expression data were not previously available, into these groups.

### Identifying Candidate Virulence Factors

Because expression of virulence-associated genes is tightly regulated (4), measuring a pathogen's gene expression in microenvironments specific to the pathogen and germane to the disease process is critical. Exploration of pathogen gene expression in the host environment may be technically challenging because of the relatively small number of pathogens present in an infected animal (29). Until more sensitive detection protocols are developed, examining global gene expression will be more practical in environmental conditions that mimic aspects of the host environment, such as elevated temperature, iron limitation, and changes in pH (4, 63) and in cell culture models. In fact, a microarray has been used to monitor gene expression in *M. tuberculosis* while it infects cultured monocytes (64). Even after measurement of bacterial gene expression from infected hosts becomes feasible, the ex vivo datasets will facilitate deconstruction of the in vivo gene expression response into component responses, leading to detailed understanding of the pathways of virulence factor regulation.

Identifying candidate virulence factors through a global gene expression method relies on two assumptions. First, because virulence-associated genes are often coordinately regulated (4), new virulence factors are likely to be coregulated with known ones. By clustering gene expression profiles across a large number of conditions, we can precisely monitor coregulation, thus revealing subtleties of regulation and leading to the identification of bona fide regulons. Second, because virulence-associated genes are tightly regulated (4), genes that are specifically expressed during infection or under conditions mimicking infection are candidate virulence factors. This assumption has been justified by numerous studies using in vivo expression technology (IVET) and differential fluorescence induction (DFI), in which genes induced during infection are often required for virulence (4, 65). When RNA from in vivo microbial samples can be efficiently isolated and labeled, microarrays will provide substantial advantages over IVET and

DFI technologies for identifying putative virulence factors, including immediate identification of differentially expressed genes and detection of temporal profiles of transcription induction and repression. As is demanded for candidate genes identified by any expression screening approach, a role in pathogenesis must be confirmed by mutation and subsequent assays of virulence.

By identifying factors expressed in the host, microarray methods may also identify potential vaccine targets. Furthermore, one could identify candidate epitopes for vaccine development for intracellular pathogens by predicting whether genes that are preferentially expressed inside host leukocytes will encode promiscuous human leukocyte antigen class II ligands (66).

Gene expression studies may also reveal key regulatory differences that lead to differing virulence between closely related pathogen strains. For example, variations in virulence of *Listeria monocytogenes* serotypes have been correlated with differential transcription of PrfA-regulated virulence genes (67, 68). However, because microarrays cannot measure expression of genes that are absent from the reference strain, genotypic differences such as horizontal transfer of virulence factors will not be detectable by this method.

### Pharmacogenomics

Yet another application for microarrays is the study of drug effects on microbial cellular physiology, as revealed by global gene expression patterns (69). This approach has been used to identify drug-specific gene expression signatures in yeast and human cells (18,19,70). Correlation of gene expression with drug activity may suggest molecular details of drug action, and correlation of transcription profiles in untreated cells with drug response may reveal mechanisms for sensitivity and resistance (19).

This approach has recently been used to characterize gene expression response in *M. tuberculosis* exposed to known inhibitors of the mycolic acid biosynthesis pathway, isoniazid and ethionamide (30). Both of these compounds elicited a similar gene expression response profile, characterized by pronounced transcription induction of five adjacent genes encoding fatty acid biosynthesis enzymes. Because a proven isoniazid target, KasA, was among these genes, the authors proposed that the adjacent, coregulated loci might be targets for new anti-tuberculosis drugs. Finally, these results suggested that the mode of action of a novel compound may be inferred from gene expression response to that compound.

Using microarrays to detect microbial polymorphisms linked to known drug-resistance phenotypes will also influence diagnosis and subsequent drug treatment. For example, an oligonucleotide array was used to detect mutant alleles of the *M. tuberculosis rpoB* gene, which are known to confer resistance to rifampicin (71).

### Microbial Genotyping

One microarray application that interrogates DNA rather than RNA is the identification of genomic deletions in mutant strains and environmental isolates by measuring the number of DNA copies at each locus, a technique termed array-based comparative genome hybridization (72). This technique was used to identify several large deletions in a number of BCG vaccine strains and reconstruct their phylogeny (73).

Oligonucleotide arrays have also been used for fine-scale genotyping of polymorphisms in related pathogens. Accurate identification of *Mycobacterium* species using a GeneChip containing a set of 82 polymorphic oligonucleotides from the 16S ribosomal RNA gene demonstrated the potential power of this approach for molecular diagnostics (71). As additional microbial genome ORF microarrays become available, molecular surveys of the genomic structure of multiple strains will become far more precise and feasible. Two caveats should be mentioned: the ability to characterize genome insertions relative to the reference sequence is lacking, and the degree to which sequence variability can be characterized on the basis of microarray hybridization is unknown.

### Examining a Host: Application of DNA Microarrays

### Designing Microarrays for Host Organisms

The currently described human DNA microarrays are largely composed of expressed sequence tags (ESTs). Culling ESTs from many different tissue sources and limiting representation of any single Unigene cluster (see http://www.ncbi.nlm.nih.gov/UniGene/Hs.stats.shtml) have resulted in better than 50% representation of the predicted 80,000-100,000 human coding regions (28). A variety of human DNA and oligonucleotide microarrays are available commercially (e.g., Incyte, Palo Alto, CA; Affymetrix; NEN Life Science Products, Boston, MA).

For in vivo studies of host response, infection of animal models will often be necessary. If the animal is a primate, human DNA microarrays might be used to monitor host gene expression because of the high level of primary sequence similarity between species. Sequence similarity is too low to permit reliable cross-hybridization with nonprimate vertebrates, but microarrays composed of mouse and rat sequences have been described (74) and are available (e.g., Incyte, Affymetrix).

### Understanding Pathogenesis

Microarrays promise to accelerate our understanding of the host side of the host-pathogen interaction. A large fraction of the genome can be simultaneously interrogated, and clustering of the data may identify groups of genes that implicate activation or repression of key regulatory pathways. Microarrays also allow the temporal sequence of transcription induction and repression to be followed, a prerequisite for determining the order of events following an encounter. Finally, ascertainment of the host cell's physiologic state, particularly apoptosis and necrosis, by genomewide profiling will facilitate separation of primary and secondary effects.

One important caveat of studying transcription in any system is that post-transcription regulatory events cannot be detected. This is particularly important in the case of host response because many important host cell events, such as cytoskeletal rearrangements, occur after transcription (75). Therefore, some key aspects of the molecular program may not be easily characterized by gene expression profiling. Eventually, it may be possible to monitor simultaneously the levels, activities, and interactions of all proteins in the cell (76).

Although analyzing gene expression of infected tissues is feasible, cellular heterogeneity may make analysis of host response complicated. Examining the response in infected cultured cells by using cell types most likely to encounter the pathogen may reduce the complexity of the system being examined. Results obtained in cell culture systems will be instrumental in interpreting gene expression profiles of specific cell types from whole tissue datasets.

The first application of global gene expression methods to pathogenesis used oligonucleotide arrays to monitor gene expression in primary human fibroblasts infected by human CMV (37). The transcript abundance of 258 out of 6,600 human genes changed by more than fourfold compared to uninfected cells at either 8 or 24 hours after infection. Some of these changes, such as induction of cytokines, stress-inducible proteins, and many interferon-inducible genes, were consistent with induction of cellular immune responses.

A similar experimental design has been used to examine the global effects of HIV-1 infection on cultured CD4-positive T cells. One study concluded that HIV-1 infection resulted in differential expression of 20 of the 1,506 human genes monitored and that most of these changes occurred only after 3 days in culture (36). In contrast, the preliminary results of an independent study using a similar design indicated that substantial HIV-induced transcription changes began very early after inoculation (77). The latter study confirmed activation of nuclear factor-κB (NF-κB), p68 kinase, and RNase L.

DNA expression arrays have recently been used to examine the response of host cells to infection by bacterial pathogens. Transcription profiling of macrophages and epithelial cells infected by *Salmonella* confirmed increased expression of many proinflammatory cytokines and chemokines, signaling molecules, and transcription activators and identified several genes previously unrecognized to be regulated by infection (33,34). The macrophage study demonstrated that exposure to purified *Salmonella* lipopolysaccharide resulted in a very similar response profile to whole cells and that activation of macrophages with gamma interferon before infection modified the response (34). In epithelial cells, overexpression of κB (an inhibitor of NF-κB) blocked induction of gene expression for a number of regulated genes, underscoring the importance of NF-κB in the proinflammatory response (33).

Similarly, the transcription response of human promyelocytic cells to *L. monocytogenes* infection has been determined by both oligonucleotide arrays and filter-based arrays (32). Comparison of these data with the *Salmonella* infection data suggests that the proinflammatory response is grossly conserved: in both cases many key components including interleukin-1, intercellular adhesion molecule-1, and macrophage inflammatory protein 1-β are induced. Although differences were observed between the two experiments, including induction of apoptosis-promoting genes by *Salmonella* versus induction of anti-apoptotic genes by *L. monocytogenes*, the disparities between cell lines, methods, and genes assayed in these reports make direct comparison difficult. However, we speculate that differences in pathogen virulence strategies may account for some of these differences in host response at the molecular level.

The initial reports demonstrate the potential power of using microarrays to characterize host response but also suggest that interpretation of host gene expression profiles will be challenging. For example, modulation of mRNAs encoding components of the prostaglandin E2 biosynthetic pathway suggested that CMV induced synthesis of this proinflammatory second messenger (37). The authors of this study proposed three potential explanations for this observation: this pathway could be induced by a cellular response intended to limit spread of the infection by promoting the killing of infected cells; viral regulators could induce prostaglandin E2 production to lure monocytes, which could subsequently be infected, leading to viral dissemination within the host; and these genes could be induced secondarily through induction of interleukin-1β since a similar pattern of regulation was observed in cells treated with that cytokine. Microarrays can identify interesting cellular events, but because expression patterns cannot distinguish between these mechanisms, the need for further investigation is obvious.

The experiments described above are strictly exploratory and attempt to catalog the transcription events that occur after an infection. However, expression profiling also lends itself to a more hypothesis-driven experimental design. For example, comparison of host responses to related strains of the same pathogen could explain differences in pathogenesis. In fact, comparison of gene expression in human monocytes infected by two distinct strains of Ebola virus, one infectious for humans and one not, revealed divergent transcription responses (78). Similarly, by examining responses to isogenic mutant pathogen strains lacking single virulence genes, or virulence factor-associated biologic activities, one might attribute components of the response to specific virulence attributes, which in turn might yield mechanistic insight into those virulence factors. Finally, comparing transcription responses to families of structurally related virulence factors, e.g., bacterial pore-forming toxins, may explain how pathogens expressing similar virulence factors can cause different pathologic responses.

### Diagnostic Gene Expression Profiles

Most microarray-based gene expression studies in humans have searched for genes that are differentially expressed in various pathologic states. For example, clustering gene expression profiles can classify tumors into separate molecular subtypes (12-17). In the case of diffuse large B-cell lymphoma, two distinct molecular classes exhibit substantially different survival rates, suggesting that future clinical intervention, at least in the case of cancer, could be guided by diagnostic gene expression profiling (14). Microarrays have also been used to measure the response of cultured cells to distinct external stimuli, including drugs (19) and environmental toxins (79).

How can this paradigm be applied to the diagnosis of infectious disease? In collaboration with Pat Brown (Stanford) and Lou Staudt (National Cancer Institute), we hypothesize that the unique constellation of virulence factors expressed by a specific pathogen will elicit a unique transcription response in the host (80). By extension, the cascade of events leading to inflammation and acquired immunity, including secretion of mediators and subsequent cell-cell interactions, might leave a unique trail of transcription signatures in the leukocytes participating in that response. Despite conserved overall virulence strategies, microbial pathogens exhibit specialization and unique attributes for any given strategy at the molecular level (81). Thus, by measuring the aggregate gene expression pattern in peripheral blood mononuclear leukocytes, for example, we may find signatures diagnostic of infection by specific pathogens or categories of pathogens.

The potential advantages of using host gene expression signatures as diagnostic markers of infection are profound. First, this technique might permit early detection of exposure to pathogens, even uncultivatable or uncharacterized pathogens. Second, variations in host signatures could be used to infer time since exposure. Third, because host response may continue in the absence of the pathogen, this method might detect exposure to pathogens that only transiently colonize the host, are sequestered in poorly accessed anatomic sites, or do not colonize the host at all (e.g., *Clostridium botulinum* and *C. perfringens*, in some cases). Finally, a single, easily collected sample could be used for diagnosing exposure to a wide array of agents.

Before the proposed method becomes an accepted diagnostic tool, one must determine whether exposure to a pathogen leads to a robust, persistent, and specific gene expression signature in peripheral blood mononuclear leukocytes and whether this signature is universal in patients of different genetic backgrounds. Experiments are under way in our laboratory to assess the feasibility of this approach. Thus far, identification of gene expression profiles common to many different pathogens is leading to a more detailed understanding of early events in the development of immune response, and inflammation in particular, but the goal of these experiments (to define unique signatures for each pathogen) has not yet been realized.

### Conclusion: The Two-Way Conversation

The few published studies reviewed here represent what is certain to be the beginning of a deluge of genome-scale pathogen data. At Stanford University alone, microarray-based studies of *Bordetella pertussis*, *Salmonella*, *H. pylori*, *Campylobacter jejuni*, *V. cholerae*, *M. tuberculosis*, and *E. coli*, as well as the nonpathogenic microbes *Streptomyces coelicolor* and *C. crescentus*, are under way (S. Falkow, G. Schoolnik, S. Cohen, and L. Shapiro, pers. comm.).

The longer term goals of functional genomics and microarray technology in infectious diseases include describing the host-pathogen interaction in molecular detail and identifying critical target molecules and pathways for diagnosis and intervention. Realizing these goals will require additional technology, extensive data collection, sophisticated computational tools, and efforts to discern cause and effect. We are on the verge of being able to listen to the two-way conversation between pathogen and host through devices of immense power.

### Acknowledgments

We gratefully acknowledge P. Brown, S. Falkow, L. Shapiro, S. Cohen, G. Schoolnik, and members of the Brown, Falkow, and Relman laboratories for helpful discussions. J. Boldrick and A. Alizadeh kindly provided illustrations.

This work was supported by grants from the Defense Advanced Research Projects Agency (#N65236-99-1-5428) and the Department of Veterans Affairs REAP Program.

Dr. Cummings is a postdoctoral fellow in the Department of Microbiology & Immunology at Stanford University, Stanford, California. His interests include *Bordetella* molecular pathogenesis and gene expression bioinformatics.

Dr. Relman is assistant professor of microbiology, immunology, and medicine at Stanford University. His interests are in the development and use of molecular methods for pathogen discovery, human microbial ecology, genomewide host and microbial responses to infection, and *Bordetella* pathogenesis.

### References

1. Blattner FR, Plunkett G, 3rd, Bloch CA, Perna NT, Burland V, Riley M, et al. The complete genome sequence of *Escherichia coli* K-12. Science 1997; 277:1453-74.
2. Carulli JP, Artinger M, Swain PM, Root CD, Chee L, Tulig C, et al. High throughput analysis of differential gene expression. J Cell Biochem Suppl 1998; 30-31:286-96.
3. Svanborg C, Godaly G, Hedlund M. Cytokine responses during mucosal infections: role in disease pathogenesis and host defence. Current Opinion in Microbiology 1999; 2:99-105.
4. Cotter PA, Miller JF. In vivo and ex vivo regulation of bacterial virulence gene expression. Current Opinion in Microbiology 1998; 1:17-26.
5. Schena M, Shalon D, Davis RW, Brown PO. Quantitative monitoring of gene expression patterns with a complementary DNA microarray. Science 1995;270:467-70.
6. Spellman PT, Sherlock G, Zhang MQ, Iyer VR, Anders K, Eisen MB, et al. Comprehensive identification of cell cycle-regulated genes of the yeast *Saccharomyces cerevisiae* by microarray hybridization. Mol Biol Cell 1998;9:3273-97.
7. Cho RJ, Campbell MJ, Winzeler EA, Steinmetz L, Conway A, Wodicka L, et al. A genome-wide transcriptional analysis of the mitotic cell cycle. Mol Cell 1998;2:65-73.
8. Chu S, DeRisi J, Eisen M, Mulholland J, Botstein D, Brown PO, et al. The transcriptional program of sporulation in budding yeast. Science 1998;282:699-705.
9. DeRisi JL, Iyer VR, Brown PO. Exploring the metabolic and genetic control of gene expression on a genomic scale. Science 1997;278:680-6.
10. Tao H, Bausch C, Richmond C, Blattner FR, Conway T. Functional genomics: expression analysis of *Escherichia coli* growing on minimal and rich media. J Bacteriol 1999;181:6425-40.

11. Richmond CS, Glasner JD, Mau R, Jin H, Blattner FR. Genome-wide expression profiling in *Escherichia coli* K-12. Nucleic Acids Res 1999;27:3821-35.
12. Khan J, Simon R, Bittner M, Chen Y, Leighton SB, Pohida T, et al. Gene expression profiling of alveolar rhabdomyosarcoma with cDNA microarrays. Cancer Res 1998; 58:5009-13.
13. Perou CM, Jeffrey SS, van de Rijn M, Rees CA, Eisen MB, Ross DT, et al. Distinctive gene expression patterns in human mammary epithelial cells and breast cancers. Proc Natl Acad Sci U S A 1999;96:9212-7.
14. Alizadeh AA, Eisen MB, Davis RE, Ma C, Lossos IS, Rosenwald A, et al. Distinct types of diffuse large B-cell lymphoma identified by gene expression profiling. Nature 2000; 403:503-11.
15. Golub TR, Slonim DK, Tamayo P, Huard C, Gaasenbeek M, Mesirov JP, et al. Molecular classification of cancer: class discovery and class prediction by gene expression monitoring. Science 1999; 286:531-7.
16. Alon U, Barkai N, Notterman DA, Gish K, Ybarra S, Mack D, et al. Broad patterns of gene expression revealed by clustering analysis of tumor and normal colon tissues probed by oligonucleotide arrays. Proc Natl Acad Sci U S A 1999;96:6745-50.
17. Wang K, Gan L, Jeffery E, Gayle M, Gown AM, Skelly M, et al. Monitoring gene expression profile changes in ovarian carcinomas using cDNA microarray. Gene 1999; 229:101-8.
18. Marton MJ, DeRisi JL, Bennett HA, Iyer VR, Meyer MR, Roberts CJ, et al. Drug target validation and identification of secondary drug target effects using DNA microarrays. Nat Med 1998;4:1293-301.
19. Scherf U, Ross DT, Waltham M, Smith LH, Lee JK, Tanabe L, et al. A gene expression database for the molecular pharmacology of cancer. Nat Genet 2000;24:236-244.
20. Southern E, Mir K, Shchepinov M. Molecular interactions on microarrays. Nat Genet 1999;21:5-9.
21. Brown PO, Botstein D. Exploring the new world of the genome with DNA microarrays. Nat Genet 1999;21:33-7.
22. Ramsay G. DNA chips: state-of-the art. Nat Biotechnol 1998;16:40-4.
23. Watson A, Mazumder A, Stewart M, Balasubramanian S. Technology for microarray analysis of gene expression. Curr Opin Biotechnol 1998;9:609-14.
24. Fodor SP, Rava RP, Huang XC, Pease AC, Holmes CP, Adams CL. Multiplexed biochemical assays with biological chips. Nature 1993;364:555-6.
25. Lockhart DJ, Dong H, Byrne MC, Follettie MT, Gallo MV, Chee MS, et al. Expression monitoring by hybridization to high-density oligonucleotide arrays. Nat Biotechnol 1996; 14:1675-80.
26. Cheung VG, Morley M, Aguilar F, Massimi A, Kucherlapati R, Childs G. Making and reading microarrays. Nat Genet 1999;21:15-9.
27. Duggan DJ, Bittner M, Chen Y, Meltzer P, Trent JM. Expression profiling using cDNA microarrays. Nat Genet 1999; 21:10-4.
28. Lipshutz RJ, Fodor SP, Gingeras TR, Lockhart DJ. High density synthetic oligonucleotide arrays. Nat Genet 1999;21:20-4.
29. de Saizieu A, Certa U, Warrington J, Gray C, Keck W, Mous J. Bacterial transcript imaging by hybridization of total RNA to oligonucleotide arrays. Nat Biotechnol 1998; 16:45-8.
30. Wilson M, DeRisi J, Kristensen HH, Imboden P, Rane S, Brown PO, et al. Exploring drug-induced alterations in gene expression in *Mycobacterium tuberculosis* by microarray hybridization. Proc Natl Acad Sci U S A 1999;96:12833-8.
31. Talaat AM, Hunter P, Johnston SA. Genome-directed primers for selective labeling of bacterial transcripts for DNA microarray analysis. Nat Biotechnol 2000;18:679-682.
32. Cohen P, Bouaboula M, Bellis M, Baron V, Jbilo O, Poinot-Chazel C, et al. Monitoring cellular responses to *Listeria monocytogenes* with oligonucleotide arrays. J Biol Chem 2000; 275:11181-90.
33. Eckmann L, Smith JR, Housley MP, Dwinell MB, Kagnoff MF. Analysis by high density cDNA arrays of altered gene expression in human intestinal epithelial cells in response to infection with the invasive enteric bacteria salmonella. J Biol Chem 2000;275:14084-94.
34. Rosenberger CM, Scott MG, Gold MR, Hancock RE, Finlay BB. *Salmonella* Typhimurium infection and lipopolysaccharide stimulation induce similar changes in macrophage gene expression. J Immunol 2000;164:5894-904.
35. Iyer VR, Eisen MB, Ross DT, Schuler G, Moore T, Lee JCF, et al. The transcriptional program in the response of human fibroblasts to serum. Science 1999;283:83-7.
36. Geiss GK, Bumgarner RE, An MC, Agy MB, van ‘t Wout AB, Hammersmark E, et al. Large-scale monitoring of host cell gene expression during HIV-1 infection using cDNA microarrays. Virology 2000;266:8-16.
37. Zhu H, Cong JP, Mamtora G, Gingeras T, Shenk T. Cellular gene expression altered by human cytomegalovirus: global monitoring with oligonucleotide arrays. Proc Natl Acad Sci U S A 1998;95:14470-5.
38. Bassett DE, Jr., Eisen MB, Boguski MS. Gene expression informatics--it’s all in your mine. Nat Genet 1999;21:51-5.
39. Khan J, Bittner ML, Chen Y, Meltzer PS, Trent JM. DNA microarray technology: the anticipated impact on the study of human disease. Biochim Biophys Acta 1999; 1423:M17-28.
40. Brazma A, Robinson A, Cameron G, Ashburner M. One-stop shop for microarray data. Nature 2000;403:699-700.
41. Array data go public. Nat Genet 1999;22:211-12.
42. Tamayo P, Slonim D, Mesirov J, Zhu Q, Kitareewan S, Dmitrovsky E, et al. Interpreting patterns of gene expression with self-organizing maps: methods and application to hematopoietic differentiation. Proc Natl Acad Sci U S A 1999;96:2907-12.
43. Eisen MB, Spellman PT, Brown PO, Botstein D. Cluster analysis and display of genome-wide expression patterns. Proc Natl Acad Sci U S A 1998;95:14863-8.
44. Ben-Dor A, Shamir R, Yakhini Z. Clustering gene expression patterns. J Comput Biol 1999;6:281-97.
45. Ross DT, Scherf U, Eisen MB, Perou CM, Rees C, Spellman P, et al. Systematic variation in gene expression patterns in human cancer cell lines. Nat Genet 2000;24:227-35.

46. Brown MP, Grundy WN, Lin D, Cristianini N, Sugnet CW, Furey TS, et al. Knowledge-based analysis of microarray gene expression data by using support vector machines. Proc Natl Acad Sci U S A 2000;97:262-7.
47. Gaasterland T, Bekiranov S. Making the most of microarray data. Nat Genet 2000; 24:204-6.
48. Berry MJA, Linoff G. Data mining techniques for marketing, sales and customer support. New York: John Wiley and Sons;1997.
49. Weaver DC, Workman CT, Stormo GD. Modeling regulatory networks with weight matrices. In: Altman RB, Lauderdale K, Dunker AK, Hunter L, Klein TE, editors. Biocomputing '99: Proceedings of the Pacific Symposium. River Edge (NJ): World Scientific Press;1999:112-23.
50. Chen T, He HL, Church GM. Modeling gene expression with differential equations. In: Altman RB, Lauderdale K, Dunker AK, Hunter L, Klein TE, editors. Biocomputing '99: Proceedings of the Pacific Symposium. River Edge (NJ): World Scientific Press; 1999:29-40.
51. Davidson GS, Hendrickson B, Johnson DK, Meyers CE, Wylie BN. Knowledge mining with VxInsight: discovery through interaction. Journal of Intelligent Information Systems, Integrating Artificial Intelligence and Database Technologies 1998;11:259-85.
52. Delcher AL, Harmon D, Kasif S, White O, Salzberg SL. Improved microbial gene identification with GLIMMER. Nucleic Acids Res 1999;27:4636-41.
53. Ramakrishna R, Srinivasan R. Gene identification in bacterial and organellar genomes using GeneScan. Comput Chem 1999;23:165-74.
54. Hayes WS, Borodovsky M. How to interpret an anonymous bacterial genome: machine learning approach to gene identification. Genome Res 1998;8:1154-71.
55. Audic S, Claverie JM. Self-identification of protein-coding regions in microbial genomes. Proc Natl Acad Sci U S A 1998; 95:10026-31.
56. Lukashin AV, Borodovsky M. GeneMark.hmm: new solutions for gene finding. Nucleic Acids Res 1998;26:1107-15.
57. Borodovsky M, McIninch J. Recognition of genes in DNA sequence with ambiguities. Biosystems 1993;30:161-71.
58. Rozen S, Skaletsky HJ. Primer3; 1996, 1997,1998. Cambridge (MA): Whitehead Institute. Code available at http://www-genome.wi.mit.edu/genome_software/other/primer3.html.
59. Hayward RE, Derisi JL, Alfadhli S, Kaslow DC, Brown PO, Rathod PK. Shotgun DNA microarrays and stage-specific gene expression in *Plasmodium falciparum* malaria. Mol Microbiol 2000;35:6-14.
60. Koonin EV, Mushegian AR, Galperin MY, Walker DR. Comparison of archaeal and bacterial genomes: computer analysis of protein sequences predicts novel functions and suggests a chimeric origin for the archaea. Mol Microbiol 1997;25:619-37.
61. Tatusov RL, Galperin MY, Natale DA, Koonin EV. The COG database: a tool for genome-scale analysis of protein functions and evolution. Nucleic Acids Res 2000; 28:33-6.
62. Chambers J, Angulo A, Amaratunga D, Guo H, Jiang Y, Wan JS, et al. DNA microarrays of the complex human cytomegalovirus genome: profiling kinetic class with drug sensitivity of viral gene expression. J Virol 1999;73:5757-66.
63. Mekalanos JJ. Environmental signals controlling expression of virulence determinants in bacteria. J Bacteriol 1992;174:1-7.
64. Mangan JA, Monahan IM, Wilson MA, Schnappinger D, Schoolnik GK, Butcher PD. The expression profile of *Mycobacterium tuberculosis* infecting the human monocytic cell line THP-1 using whole genome microarray analysis. Nat Genet 1999;23:61.
65. Chiang SL, Mekalanos JJ, Holden DW. In vivo genetic analysis of bacterial virulence. Annu Rev Microbiol 1999;53:129-54.
66. Sturniolo T, Bono E, Ding J, Raddrizzani L, Tuereci O, Sahin U, et al. Generation of tissue-specific and promiscuous HLA ligand databases using DNA microarrays and virtual HLA class II matrices. Nat Biotechnol 1999;17:555-61.
67. Sokolovic Z, Schuller S, Bohne J, Baur A, Rdest U, Dickneite C, et al. Differences in virulence and in expression of PrfA and PrfA-regulated virulence genes of *Listeria monocytogenes* strains belonging to serogroup 4. Infect Immun 1996; 64:4008-19.
68. Bohne J, Kestler H, Uebele C, Sokolovic Z, Goebel W. Differential regulation of the virulence genes of *Listeria monocytogenes* by the transcriptional activator PrfA. Mol Microbiol 1996;20:1189-98.
69. Debouck C, Goodfellow PN. DNA microarrays in drug discovery and development. Nat Genet 1999;21:48-50.
70. Gray NS, Wodicka L, Thunnissen AM, Norman TC, Kwon S, Espinoza FH, et al. Exploiting chemical libraries, structure, and genomics in the search for kinase inhibitors. Science 1998;281:533-8.
71. Troesch A, Nguyen H, Miyada CG, Desvarenne S, Gingeras TR, Kaplan PM, et al. *Mycobacterium* species identification and rifampin resistance testing with high-density DNA probe arrays. J Clin Microbiol 1999;37:49-55.
72. Pollack JR, Perou CM, Alizadeh AA, Eisen MB, Pergamenschikov A, Williams CF, et al. Genome-wide analysis of DNA copy-number changes using cDNA microarrays. Nat Genet 1999;23:41-6.
73. Behr MA, Wilson MA, Gill WP, Salamon H, Schoolnik GK, Rane S, et al. Comparative genomics of BCG vaccines by whole-genome DNA microarray. Science 1999;284:1520-3.
74. Khan J, Bittner ML, Saal LH, Teichmann U, Azorsa DO, Gooden GC, et al. cDNA microarrays detect activation of a myogenic transcription program by the PAX3-FKHR fusion oncogene. Proc Natl Acad Sci U S A 1999;96:13264-9.
75. Finlay BB, Falkow S. Common themes in microbial pathogenicity revisited. Microbiol Mol Biol Rev 1997;61:136-69.
76. Uetz P, Giot L, Cagney G, Mansfield TA, Judson RS, Knight JR, et al. A comprehensive analysis of protein-protein interactions in *Saccharomyces cerevisiae.* Nature 2000; 403:623-7.

77. Corbeil J, Sheeter D, Rought S, Du P, Ferguson M, Masys DR, et al. Magnitude and specificity of temporal gene expression during HIV-1 infection of a CD4+ T cell. Nat Genet 1999;23:39-40.
78. Xiang C, Young H, Alterson H, Reynolds D, Bittner M, Chen Y, et al. Comparison of cellular gene expression in Ebola-Zaire and Ebola-Reston virus-infected primary human monocytes. Nat Genet 1999;23:82.
79. Nuwaysir EF, Bittner M, Trent J, Barrett JC, Afshari CA. Microarrays and toxicology: the advent of toxicogenomics. Mol Carcinog 1999;24:153-9.
80. Roebuck KA, Carpenter LR, Lakshminarayanan V, Page SM, Moy JN, Thomas LL. Stimulus-specific regulation of chemokine expression involves differential activation of the redox-responsive transcription factors AP-1 and NF-kappaB. J Leukoc Biol 1999; 65:291-8.
81. Lo D, Feng L, Li L, Carson MJ, Crowley M, Pauza M, et al. Integrating innate and adaptive immunity in the whole animal. Immunol Rev 1999;169:225-39.
